# Supplementary material for: Liposomal Formulation of Botanical Extracts may Enhance Yield Triggering PR Genes and Phenylpropanoid Pathway in Barley (Hordeum vulgare)
Source: Plants (Basel). 2022 Nov 3;11(21):2969. doi: 10.3390/plants11212969 (PMC9658110; doi:10.3390/plants11212969)
Supplement: Supplementary file 1 [file plants-11-02969-s001.zip › plants-1976972-supplementary.pdf]

Supplemental Materials

Supplemental Table S1. Characteristics of field experiments of *Hordeum vulgare* test crop.

Examined culture	
Species name (English/Latin)	winter barley / *Hordeum vulgare*
Breed	SU Ellen
Sowing time	13.10.2020.
Number of plants	4.5 million germs / ha
Row spacing	12 cm
Planting distance:	1.2 cm
Other stock characteristics	homogeneous stock
Harvest time	12/07/2021
**Experimental site**	
County, locality, factory:	Komárom-Esztergom County, Tata, Hungary, EOV 592699 255447
Elevation	144 m
Soil physical variety	Sandy loam
pH (KCl)	7.4
Commitment (CA)	34
Humus content (%)	1.77
CaCO3 (w / w%)	11
CEC	18.5
Pre-crop	spring barley
Plot size	75 m2
Number of repetitions	4

Supplemental Table S2. Technical data of treatment of Elice16Indures in field experiments.

Treatment	A	B
Treatment time	05/05/2021	17/05/2021
Crop Phenology	BBCH 39	BBCH 51
Meteorological conditions		
air temperature, ° C	17.2 ° C	23.3 ° C
rel. humidity,%	63.40%	74.50%
cloud cover,%	25%	75%
Application technical data		
Spraying	TTAM4E spraying drone	TTAM4E spraying drone
Spray juice (l / ha)	50 l / ha	50 l / ha
Spraying method	stock spraying	stock spraying
Dosage	10-240g/ha	10-240g/ha

Supplemental Table S3. Determination of crop yield calculating statistical data of four parallel parcels (kg plots $^{-1}$).

Treatment	Dose g*ha-1	Yield kg/plots (1.10*8)					
		I	II	III	IV	average	control %
control	control	4,4	4,8	5,1	4,8	4,775	100
Fitokondi	Fitokondi(4l*ha-1)	5,2	5,2	4,1	6,1	5,15	107,9
Elice16Indures	10	6,9	6,9	5,3	5,2	6,075	127,2
Elice16Indures	20	5,6	4,6	4,5	6,5	5,3	111
Elice16Indures	30	4,3	4,2	6,2	6,2	5,225	109,4
Elice16Indures	60	5,3	5,8	6,5	4,9	5,625	117,8
Elice16Indures	120	4,8	5,7	4,4	6,4	5,325	111,5
Elice16Indures	240	6,8	6,8	6,8	6,2	6,65	139,3
SzD10%						0,975	20,4
SzD5%						1,176	24,6
SzD1%						1,593	33,4
variancia							
G=	176,5						
C=	973,5078125						
	SQ	FG	MSQ	Fsz	Ftábl.	T-ért.	SzD
All	25,4221875						
Repeat	0	0			1,98262518	1,71088208	0,974649218
Treatment	9,8446875	7	1,406383929	2,166792764	2,422628533	2,063898562	1,175754392
Error	15,5775	24	0,6490625		3,49592752	2,796939505	1,593350549

Supplemental Table S4. Determination of moisture calculating statistical data of four parallel parcels (%).

Treatment	Dose g*ha-1	Moisture%					
		I	II	III	IV	average	control %
control	control	8,82	9,08	9,03	8,01	8,735	100
Fitokondi	Fitokondi, 4l*ha-1	9,06	8,02	9,09	9,04	8,803	100,8
Elice16Indures	10	8,09	9,05	8,07	9,06	8,568	98,1
Elice16Indures	20	8,06	9,06	8,09	8,06	8,318	95,2
Elice16Indures	30	8,02	9,01	8,02	9,04	8,523	97,6
Elice16Indures	60	8,03	9,04	9,01	8,07	8,538	97,7
Elice16Indures	120	8,01	8,02	9,04	8,09	8,29	94,9
Elice16Indures	240	9,06	8,07	8,03	8,02	8,295	95
SzD10%						0,641	7,3
SzD5%						0,773	8,9
SzD1%						1,048	12
variancia							
G=	272,27						
C=	2316,592278						
	SQ	FG	MSQ	Fsz	Ftábl.	T-ért.	SzD
All	7,829221875						
Repeat	0	0			1,98262518	1,71088208	0,641151728
Treatment	1,088246875	7	0,155463839		2,422628533	2,063898562	0,77344438
Error	6,740975	24	0,280873958	0,553500368	3,49592752	2,796939505	1,048150903

Supplemental Table S5. Determination of crop yield at 8% moisture content, calculating statistical data of four parallel parcels (T ha $^{-1}$).

Treatment	Dose g*ha-1	Yield t*ha-1 (at 8% moisture content)					
		I	II	III	IV	average	control %
control+I2B4:H8	control	3,96	4,31	4,58	4,36	4,306	100
Fitokondi	Fitokondi (4l*ha-1)	4,67	4,73	3,68	5,48	4,641	107,8
Elice16Indures	10	6,27	6,2	4,81	4,67	5,489	127,5
Elice16Indures	20	5,09	4,13	4,09	5,91	4,803	111,5
Elice16Indures	30	3,91	3,78	5,64	5,57	4,723	109,7
Elice16Indures	60	4,82	5,21	5,84	4,45	5,081	118
Elice16Indures	120	4,36	5,18	3,95	5,81	4,828	112,1
Elice16Indures	240	6,11	6,18	6,18	5,64	6,026	139,9
SzD10%						0,883	20,5
SzD5%						1,065	24,7
SzD1%						1,443	33,5
variancia							
G=	159,5889032						
C=	795,8943129						
	SQ	FG	MSQ	Fsz	Ftábl.	T-ért.	SzD
All	20,98641922						
Repeat	0	0			1,98262518	1,71088208	0,8828338
Treatment	8,20559131	7	1,17222733	2,201223279	2,422628533	2,063898562	1,064994153
Error	12,78082791	24	0,532534496		3,49592752	2,796939505	1,443251269

Supplemental Table S6. The active compounds of botanical extracts used to produce ELICE16INDURES.

Latin binomial name	Plant part	Main active compounds
Urtica dioica	leaves	phenolic ingredients, caffeic acid, malic acid
Melissa officinalis	leaves	citral, caryophyllene, citronellal, geranyl acetate, caryophyllene oxide
Carum carvi	seeds	carvone, limonene
Cinnamon Ceylanicum	bark	cinnamaldehyde, o-methoxy cinnamaldehyde, coumarin
Syzygium aromaticum	flower bud	eugenol, caryophyllene, eugenyl acetate
Allium sativum	garlic cloves	diallyl disulfide, diallyl trisulfide, methyl allyl disulfide, vinyldithiine, ajoene, allicin
Zingiber officinalis	rhizome	zingiberene, farnesene, curcumene
Calendula officinalis	flower	pentacyclic triterpene alcohols and triterpendiol monoesters, faradiol esters esterified with mainly myristic- and palmitic acids
Origanum majorana	leaves	bicyclic monoterpenes cis- and trans-sabinene hydrate
Salvia officinalis	leaves	alpha and beta thujone, camphor, cineole
Thymus vulgaris	leaves	thymol, p-cymol
